# Intraspecific perspective of phenotypic coordination of functional traits in Scots pine

**DOI:** 10.1371/journal.pone.0228539

**Published:** 2020-02-13

**Authors:** Bárbara Carvalho, Cristina C. Bastias, Adrián Escudero, Fernando Valladares, Raquel Benavides

**Affiliations:** 1 Departamento Biogeografía y Cambio Global, LINCGlobal, Museo Nacional de Ciencias Naturales, (MNCN-CSIC), Madrid, Spain; 2 Área de Biodiversidad y Conservación, Universidad Rey Juan Carlos, Móstoles, Madrid, Spain; Helmholtz Centre for Environmental Research - UFZ, GERMANY

## Abstract

Functional traits have emerged as a key to understand species responses to environmental conditions. The concerted expression of multiple traits gives rise to the phenotype of each individual, which is the one interacting with the environment and evolving. However, patterns of trait covariation and how they vary in response to environmental conditions remain poorly understood, particularly at the intraspecific scale. Here, we have measured traits at different scales and in different organs, and analysed their covariation in a large number of conspecifics distributed in two contrasting environments. We expected significant correlations among traits, not only within clusters of traits as found in global, multispecies studies, but also among clusters, with more relationships within clusters, due to genetic constraints, and among clusters due to more coordinated phenotypes than community level, multispecies studies. We surveyed 100 *Pinus sylvestris* trees in a Mediterranean mountainous area distributed in two contrasting elevations. We measured 13 functional traits, in three clusters (leaf, stem and whole-plant traits), and analysed their variation and coordination. We found significant coordination among traits belonging to different clusters that reveals coordinated phenotypes. However, we found fewer correlations within trait clusters than initially expected. Trait correlation structures (number, intensity and type of correlations among traits) differed among individuals at different elevations. We observed more correlations within trait clusters at low elevation compared to those at high elevation. Moreover, the higher number of correlations among different trait clusters and the lower trait variation at the higher elevation suggests that variability decreases under more stressful conditions. Altogether, our results reveal that traits at intraspecific scale are coordinated in a broad network and not only within clusters of traits but also that this trait covariation is significantly affected by environmental conditions.

## Introduction

One of the major challenges in plant ecology over the last decades has been the development of a general plant classification framework based on adaptive plant strategies [1,2]. The dominant species-based or taxonomic perspective of communities ecology have been gradually replaced by an approach based on functional traits, which can capture general adaptive features in a continuous framework [3]. Plant functional traits are morphological, phenological or physiological features able to confer competiveness and induce niche differences among coexisting plant species [4]. Therefore, they are considered indicators of the ecological role and realised niche of each species within plant communities [3,5,6]. Differences in functional traits among species are then associated with specific ecological strategies, competitiveness and niche breadth and shape whose study are indispensable to elucidate the mechanisms underlying the assembly of plant communities [7,8].

Relationships among functional traits in plant species define coordinated spectra related to common variations in functional strategies of plants. One of the most studied spectrum is the so-called leaf economics spectrum (LES), which predicts how leaf traits should vary across environmental gradients and co-vary among themselves. It describes a universal axis from “slow” to “fast” resource use strategies of plant species [9–13] confronting low-cost, short-lived leaves with rapid return of carbon and nutrients *vs*. costly long-lived leaves with slow returns. Similarly, the wood economics spectrum (WS) reflects the trade-offs among important wood functions like transport safety, transport efficiency, and mechanical support. Within WS, wood density works as an integrator of wood properties, related to the mechanical support and resistance to embolism in drought periods [14]. As a result of the interaction of the different traits belonging to distinct plant organs, different phenotypes are displayed at the individual level [15,16], which is likely to have a profound adaptive significance in contrasting environments [17,18].

**Fig 1:**
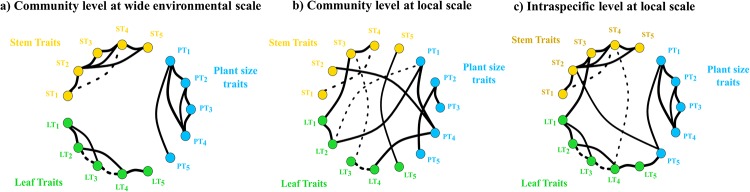
Conceptual figure with general patterns depicted from previous studies of trait correlations (either positive or negative, continuous vs. dashed lines) at different scales (from local to wide environmental spatial scale). Relationships among traits at **(a)** large scale where trait clusters (PT: plant size traits, ST: stem traits and LT: leaf traits) of numerous species are mostly independent from each other (elaborated from Baraloto et al., 2010; Fortunel et al., 2012; Díaz et al., 2016). Relationships among traits at **(b)** the community, local scale with increased level of phenotypic coordination particularly among trait clusters (elaborated from Bucci et al., 2004; Santiago et al., 2004; Ishida et al., 2008; de la Riva et al., 2016; Messier et al., 2017b). Relationships among traits **(c)** at the intraspecific, local level: we expected an intermediate situation from a) and b) with more relationships within cluster than b) due to genetic constraints, and among clusters than a) due to more integrated phenotypes.

Searching for comprehensive plant strategies, some works addressed trait covariation studies among these spectra or cluster of traits [19–23]. However, results are contrasting depending on the spatial scale of the study. For instance, studies working at wide environmental scales with multiple species showed that leaf and wood economics spectraare not coupled [20,21,24] claiming that trait covariation at wide environmental scales occurs mainly within spectra and independently among trait clusters (Fig 1a). However, Messier et al. [23] evidenced important coordination of traits from different trait clusters when the analysis is conducted at the community level, i.e. when coexisting species have overcome the same environmental filters and limitations (Fig 1b). These findings concur with many others describing relationships between WS and LES traits at the community and intraspecific scales, which showed that denser wood is positively related with more long-lasting leaves [22,25–28]. These studies suggest that traits from different clusters shape a comprehensive plant economics spectrum [29].

Intraspecific trait variation is gaining increased recognition [8, 30] and the relationships among traits within species are also expected to change in response to different environmental conditions [31,32]. Plants respond to biotic and abiotic conditions and their phenotypic expressions are bound to coordination rules acting at different spatial scales [33,34]. The number of trait combinations and therefore the number of phenotypes are not unlimited; some are constrained by genetic or biophysical factors, while others are just unsuitable for a given environment and discarded by natural selection [35]. Coordinated changes among plant organs (and traits) can be the result of natural selection that hampers one organ to evolve independently of the other [16,36].

Environmental conditions within the geographical and altitudinal distributions of a plant species vary determining important changes in the phenotypic expressions among populations [37,38]. For instance, at high elevation, plants face changes in physical conditions like temperature decrease, frost, snow accumulation, strong winds or high irradiance [39]. Accordingly, several studies demonstrated trait adjustments aligned to a ‘economic’ strategy searching for resource conservation (persistence), like increasing leaf mass per area (LMA) and carbon isotope discrimination (δ^13^C), and lower leaf area and photosynthetic pigments due to low temperatures [40,41]. Moreover, nutrients uptake limitations in cold environments affect the growth rates at high elevation, likely decreasing the leaf nitrogen content and favouring a reduction in the specific leaf area [42,43]. Evidence also showed changes in stem properties with increasing elevation, thus wood density tends to be higher [28] and bark thickness higher protecting against frost [44] compared to individuals at low elevations.

Here, we measured traits in different plant systems (hereafter, trait clusters), i.e. stem, leaf and whole-plant traits, in individual tress of *Pinus sylvestris* (Scots pine) in a Mediterranean mountainous area in Spain at two different elevations. We firstly explored the overall trait network at the intraspecific level, analysing trait correlations within and among trait clusters. Secondly, we compared trait coordination in trees at the two very close sites but with contrasting elevations, to quantify the environmental impact on trait networks. We assumed that the environmental differences between both study sites are mainly driven by elevation. We expected to find a high number of correlations among traits within trait clusters, higher than those found in multispecies studies due to the genetic constraints within a species. We also expected a large number of correlations among the three trait clusters, similarly to multispecies studies at the community level due to the existence of important relationships among clusters also at the individual level (Fig 1c).

## Material and methods

### Study area

The study was performed at the Sierra de Guadarrama National Park (Madrid, Spain) during June and July 2016. Particularly, we chose two sites very close geographically (ca 7km) but with contrasting elevations, namely Pingarrón at 1900m (40° 48´50”N; 3° 58´12”W) and Ventorrillo Biological Station at 1440m (40° 45´31”N; 4° 00´49”W). Both sites represent natural, dominant and well-conserved stands of *P*. *sylvestris* with no signs of recent management and an uneven age structure. They represent the natural highest and lowest elevational distribution of the species in the area. In both sites, the climate is mountainous Mediterranean with wet, cold winters and warm dry summers. Annual mean temperature is 7.9°C in Ventorrillo and 6.5°C in Pingarrón and annual precipitation is 897 mm in Ventorrillo and 1242 mm in Pingarrón. Mean precipitation during the three driest months is 22 mm in Pingarrón and 24 mm in Ventorrillo (*Worldclim*; [45]). The bedrock in the area is mainly composed of granite and gneiss, and soils are acid and relatively homogeneous, predominantly humic cambisol soils with leptosol at higher-elevation sites [46]. Field work was carried out in the framework of an official research grant from the Autonomous Region of Madrid (REMEDINAL TE-CM (S2018/EMT-4338) and no specific authorization for the activities included in this paper was requested.

Pingarrón site is facing north and the understorey vegetation includes oromediterrenean shrublands, such as *Juniperus communis* L. subsp alpina (Neilr.) Čelak, *Cytisus oromediterraneus* (G. López & C.E. Jarvis) Rivas Mart. and *Adenocarpus complicatus* (L.) Link. Ventorrillo site has a south-west exposure and includes a layer of deciduous Pyrenean oak (*Quercus pyrenaica* Willd.) under the dominant pine canopy, and the understorey vegetation is mainly composed of *Cistus laurifolius* L., *Cytisus scoparius* L. and *Genista cinerascens* Lange.

### Sampling design and functional trait measurements

We conducted an individual-based sampling, with 50 individuals sampled at Pingarrón (1900m) and 50 individuals at Ventorrillo (1440m). We randomly selected individuals among the mature and healthy ones and collected a fully sun-exposed branch from the top of the crown. We also extracted two 5-mm diameter wood cores at 50cm above the ground, using an increment borer.

In each tree, we measured 13 functional traits involved in relevant plant ecological functions. A summary of traits included in our data set and description of their ecological significance are described in Table 1 and related literature (S1 Table). We selected a wide variety of traits with different natures (morphological and chemical) classified in three trait clusters critical for the global plant spectrum: i) Plant size-related traits (whole-plant traits) that reflect the ability to compete for resources: plant height (m), crown depth (m) and diameter at breast height (DBH, cm); ii) Stem traits, related to transport and defence functions: bark thickness (mm) and trunk wood density (WD, g/cm^3^), and iii) Leaf traits, that balance construction costs vs. growth potential: leaf dry matter content (LDMC; mg/g), specific leaf area (SLA; mm^2^/mg), leaf nitrogen content (LNC; %), leaf carbon content (LCC; %), leaf carbon isotope (δ^13^C, ‰), chlorophyll *a* (μg/g), chlorophyll *b* (μg/g) and beta-carotene (μg/g). Detailed methods for trait measurements are provided in the Supporting Information (S1 Appendix). Before further analyses, we compared DBH, plant height and age between both populations to discard differences due to different tree size and ontogeny on traits (S2 Appendix). Note that we did not remove correlated traits, because we aimed at evaluating the phenotypic coordination (statistical integration [47]). We assumed that correlated traits exhibited either different patterns of correlation with other traits, or different patterns of change along elevation [48].

**Table 1:** Study traits and their functional significance.

Trait cluster	Trait	Ecological significance	Main Function	Abbreviation	Unit
**Plant size**	**Plant height**	Competitive ability, photosynthetic behaviour, hydraulic limitations and probability of fire escape.	**Competitive ability**		**m**
	**Crown depth**	Magnitude of light capture, competitive vigour, tree growth performance	**Competitive ability**		**m**
	**Diameter at breast height**	Competitive vigour, whole plant fecundity, growth time between disturbances, photosynthetic behaviour and probability of fire escape	**Competitive ability**	**DBH**	**cm**
**Stem traits**	**Bark thickness**	Trunk insulation against fire, pathogen, frost and drought, and trunk mechanical strength	**Mechanical support**		**mm**
	**Stem wood density**	Growth-survival trade-off, mechanical resistance, water storage in the trunk, net CO2 assimilation, hydraulic safety and response to precipitation and altitude	**Mechanical support**	**Stem WD**	**g/cm**^**3**^
**Leaf trait (Leaf morpho-anatomy)**	**Leaf dry matter content**	Structural support of the leaf, anti-herbivory resistance, leaf tissue density, leaf life-span, relative growth rate of the plant	**Resource acquisition and conservation**	**LDMC**	**mg/g**
	**Specific leaf area**	Resource acquisition, photosynthetic rate, relative growth rate of the plant, shade-tolerance	**Resource acquisition and conservation**	**SLA**	**mm**^**2**^**/mg**
**Leaf traits (Leaf chemical composition)**	**Leaf nitrogen content**	Net photosynthetic capacity, relative growth rate and N availability in the soil, leaf life-span and leaf decomposability	**Resource acquisition and conservation**	**% LNC**	**%**
	**Leaf carbon content**	Leaf palatability, leaf lignin, leaf density, relative growth rates and structural support of the leaf	**Resource acquisition and conservation**	**% LCC**	**%**
	**Carbon isotope**	Water use efficiency, the ratio of internal to atmospheric CO_2_ concentration, stomatal conductance, soil moisture, air temperature	**Resource acquisition and conservation**	**δ**^**13**^**C**	**‰**
	**Chlorophyll a**	Maximize net carbon gain; photosynthetic activity	**Resource acquisition and conservation**		**μg/g**
	**Chlorophyll b**	Maximize net carbon gain; photosynthetic activity	**Resource acquisition and conservation**		**μg/g**
	**Beta-carotene**	Maximize net carbon gain; photosynthetic activity	**Resource acquisition and conservation**		**μg/g**

### Statistical analyses

We first described the magnitude of intraspecific trait variability (ITV) for each trait by calculating the coefficient of variation (CV) and its 95% confidence limits using bootstrapping with replacement (with 500 replicates). We performed a t-test to analyse differences in trait values between sites. The traits (SLA, LNC, Chlorophyll *a*, Chlorophyll *b*, Beta-carotene, stem WD and DBH) were transformed with a Box-Cox transformation as appropriate, to meet normality assumption using *‘AID’* R package and *boxcoxnc* function.

We ran a correlation analysis to evaluate pairwise relationships among all traits, pooling data of individuals from both sites and separately. Secondly, we compared the three correlation matrices using a chi-square test. This test provides a χ^2^ value that represents the difference between a pair of correlation matrices, hypothesizing that they do not differ. Then, we also estimated how closely connected are our trait clusters from each other using network analyses. These analyses are quantitative approaches assessing the connectivity and distance of interconnected objects. The objects, our traits, are represented as nodes and their connectivity, their correlations, are represented as edges linking them. We constructed network graphs and carried out network analyses with the ‘*igraph’* package using only the statistically significant trait correlations (*p <* .*05*). We used the *modularity()* function to measure the structure of the network for both populations. This metric calculates the fraction of edges within the defined clusters minus the expected fractions if the edges were random [49,50]. High modularity (close to *Q* = 1, the maximum) reflects dense connections within a trait cluster and weak connections among clusters. If the number of connections is close to zero (*Q* = 0) the trait network has low modularity.

To assess the influence of elevation on all traits and their relationships, we performed an ordination analysis. Trait data for ordination analysis were standardized using the R function *scale*. We initially used a Detrended Correspondence Analysis (DCA), to estimate the axes length in units of average standard deviation [51,52]. As the length of the first DCA axis was relatively short (Standard Deviation units; uSD = 0.13), we conducted a Redundancy Analysis (RDA) [53], which assumes linear relationships between elevation and the elements of the trait matrix [54,55]. In our case, the trait matrix was constrained by elevation. Total variation explained (TVE) of data set was the value of canonical extracted axes (Σcons) using the constraining data matrix [56]. Finally, we performed a Monte Carlo permutation test (1000 randomizations) to determine the accuracy of the relationship between the elevation and the trait data sets.

All statistical analyses were conducted in R version 3.5.1 [57]. Comparison of trait correlation matrices between sites was performed by using the *cortest*.*mat* function in the '*psych'* package [58]. Multivariate analysis was conducted using the '*vegan'* package [59].

## Results

### Traits and their correlations

The magnitude of variation at the intraspecific level depended on the study trait (Tables 1 and 2). Both morphological (LDMC, SLA) and chemical leaf traits (%LNC, %LCC and δ^13^C) and WD exhibited relatively low intraspecific variability (ITV) with coefficients of variation (CV) below 15% (Table 2). In contrast, plant size traits (plant height, crown depth and DBH); bark thickness, photosynthetic pigments (chlorophyll *a*, *b* and beta-carotene) showed substantial variability with CV between 29% and 50% (Table 2).

**Table 2:** Descriptive statistics of the study traits measured on *Pinus sylvestris* individual trees.

	**Plant height**	**Crown depth**	**DBH**	**Bark thickness**	**Stem WD**	**LDMC**	**SLA**
Pingarrón (1900m)							
Range	2.11–20.76	1.39–16.85	4.0–107.9	0.00–69.0	0.37–0.67	304.88–467.35	4.78–8.45
Mean ± SD	13.68 ± 3.93	8.01 ± 3.51	34.4 ± 21.29	31.33 ± 13.27	0.54 ± 0.06	397.87 ± 31.42	5.91 ± 0.81
CV	0.28 (0.26, 0.30)	0.41 (0.38, 0.43)	0.47 (0.44, 0.50)	0.39 (0.34, 0.41)	0.11 (0.10, 0.12)	0.075 (0.07, 0.078)	0.13 (0.11, 0.13)
Ventorrillo (1440m)							
Range	3.61–20.08	2.89–16.18	0.53–71.0	4.67–52	0.43–0.80	317.23–489.99	3.71–7.70
Mean ± SD	12.49 ± 3.72	8.21 ± 3.61	37.40 ± 19.29	26.83 ± 10.99	0.53 ± 0.07	406.18 ± 36.16	5.44 ± 0.84
CV	0.29 (0.26, 0.30)	0.42 (0.39, 0.44)	0.53 (0.49, 0.56)	0.41 (0.38, 0.43)	0.13 (0.11, 0.14)	0.084 (0.078, 0.089)	0.15 (0.14, 0.16)
*t*-value	1.88	0.4	0.72	2.03*	0.19	2.13*	2.75**
Pooled data							
Range	2.11–20.76	1.39–16.35	0.53–107.9	0.0–69.0	0.37–0.8	304.88–489.99	3.71–8.45
Mean ± SD	12.91 ± 3.89	8.45 ± 3.56	38.05 ± 20.34	28.43 ± 12.44	0.54 ± 0.06	404.48 ± 34.71	5.75 ± 0.86
CV	0.29 (0.26, 0.31)	0.41 (0.39, 0.43)	0.50 (0.47, 0.53)	0.41 (0.37, 0.44)	0.12 (0.11, 0.13)	0.08 (0.07, 0.08)	0.14 (0.13, 0.15)
	**%LNC**	**%LCC**	**δ**^**13**^**C**	**Chlorophyll *a***	**Chlorophyll *b***	**Beta-carotene**	
Pingarrón (1900m)							
Range	1.04–2.15	47.48–53.28	-29.55 - (-25.45)	331.88–3241.68	341.95–1863.88	0.00–188.54	
Mean ± SD	1.39 ± 0.23	49.77 ± 1.04	-27.29 ± 0.95	2356.66 ± 624.39	1366.67 ± 316	134.19 ± 34.88	
CV	0.15 (0.13, 0.16)	0.021 (0.019, 0.022)	0.036 (0.033, 0.037)	0.29 (0.25, 0.31)	0.25 (0.22, 0.27)	0.31 (0.28, 0.35)	
Ventorrillo (1440m)							
Range	1.07–2.08	46.77–51.65	-30.97 - (- 24.82)	635.57–4167.86	557.01–2631.01	47.32–217.78	
Mean ± SD	1.39 ± 0.2	49.22 ± 1.24	-26.97 ± 1.18	2789.03 ± 843.45	1680.54 ± 444.28	154.43 ± 38.44	
CV	0.11 (0.11, 0.12)	0.026 (0.024, 0.026)	0.046 (0.042, 0.049)	0.29 (0.26, 0.31)	0.27 (0.25, 0.29)	0.26 (0.24, 0.28)	
*t*-value	0.26	1.92	0.55	2.50*	3.74***	2.75**	
Pooled data							
Range	1.04–2.15	46.77–53.28	-55.79	331.88–4167.86	341.94–2631.01	0.00–217.78	
Mean ± SD	1.41 ± 0.21	49.57 ± 1.16	-27.18 ± 1.07	2455.86 ± 767.88	1462.77 ± 414.09	136.20 ± 38.20	
CV	0.13 (0.12, 0.14)	0.02 (0.02, 0.03)	0.04 (0.03, 0.04)	0.30 (0.27, 0.32)	0.29 (0.27, 0.31)	0.30 (0.27, 0.32)	

Confidence limits surrounding coefficients of variation (CV) were calculated through bootstrapping with replacement (with n = 500 replicates). Comparison of traits values at the two different elevations using a t-test is also shown (*p < .05; **p < .01; ***; *p* < .0001).

**Fig 2:**
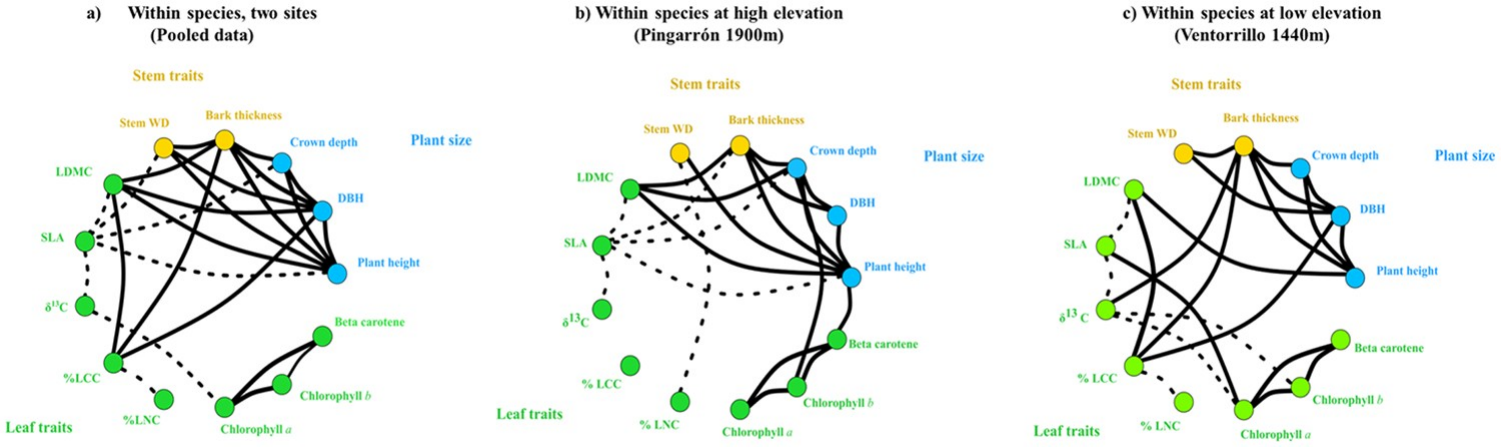
Correlograms of traits measured in individual Scots pines calculated with pooled data and data from both study sites separately (Pingarrón at 1900m and Ventorrillo at 1440m). Solid lines represent positive correlations, and dashed lines negative correlations. Line thicknesses are proportional to the correlation strength. Only significant correlations are shown. Plant size traits: plant height, crown depth and DBH: Stem traits: WD and bark thickness; Leaf traits: LDMC, SLA, δ^13^C, %LCC, %LNC, Chlorophyll *a*. Chlorophyll *b* and Beta-carotene. Different colours indicate different trait clusters and their different functions (yellow: stem traits, primarily mechanical support; blue: plant size traits, primarily competitive ability; green: leaf traits, primarily resource acquisition and conservation).

The correlation structure with pooled data had an edge density of 32% of the total pairwise correlations (Fig 2a; S2a Table). We found positive significant correlations within trait clusters, i.e. among the three architectural traits (plant height, DBH and crown depth) and between the two stem traits (WD and bark thickness). Regarding leaf cluster, the results showed some correlations describing the trade-off between acquisition (SLA, %LCN, Chlorophyll *a*) and conservation (LDMC, %LCC, δ^13^C) of resources (Fig 2a; S2a Table). The three photosynthetic pigments were positively correlated among them, without any other connection with other leaf trait.

We found also correlations among clusters. Plant size traits and stem traits were positively correlated (Fig 2a; S2a Table). Among leaf traits, LDMC was positively correlated to plant height, DBH and bark thickness; leaf carbon content (%LCC) increased with increasing DBH and bark thickness, and SLA correlated negatively with WD, crown and plant size (crown depth and plant height) (Fig 2a; S2a Table). Modularity test for pooled data showed that the study traits presented more connections within clusters than across them (*Q* = 0.10).

### Elevation effect on traits and their correlations

The results from RDA ordination showed that elevation accounted for a low and significant fraction of the total variation explained (5%) when all traits were simultaneously considered (S2 Fig; Σcons = 0.64; TVE = 0.049; Monte Carlo test: *F*_*1*,*87*_ = 4.56; *p* = 0.001). The first RDA axis represents the traits which differed between the two sites, i.e. chlorophyll *a*, LDMC and SLA. Despite the small fraction of the total variance explained by altitudinal condition, some leaf trait values were significantly different between both sites (SLA, LDMC and photosynthetic pigments) and bark thickness (Table 2). Particularly, individuals at high elevation presented a significantly greater bark thickness and higher SLA; while individuals at low elevations showed a significantly higher LDMC and photosynthetic pigments content (Table 2; S1 Fig).

The correlation analysis independently run for each site showed a similar number of significant trait correlations, 21 for individuals at high-elevation (26% of the total pairwise correlations tested) and 22 for individuals at low-elevation (28%). We also found a greater number of trade-offs, significant negative correlations, among different clusters at high elevation (13) compared with low elevation (8) (Fig 2b and 2c; S2b Table). Results from the modularity test reflected that traits at high elevation (*Q* = 0.0034) have denser connections among clusters and sparse connections within clusters than traits at low elevation (*Q* = 0.23).

Correlations between plant size and stem traits were strong and similar for individuals at different elevations. Results also showed similar patterns with leaf traits for both sites, namely a positive correlation among leaf photosynthetic pigments, and a negative correlation between SLA and LDMC and between SLA and δ^13^C (Fig 2b and 2c; S2b Table). However, we found some different outcomes between both elevations, resulting in different correlation matrices (χ^2^ = 238.38, df = 156, *p* < 0.00001). For instance, at high elevation, we found that SLA was negatively (and LDMC positively) correlated with bark thickness, crown depth and plant height. Moreover, chlorophyll *b* was positively related to crown depth and beta-carotene showed a positive correlation with plant height (Fig 2b; S2b Table). Among all significant correlations found at low elevation, SLA was positively related with chlorophyll *a*, and LDMC was positively related with %LCC and plant height. Photosynthetic pigments, chlorophyll *a* and *b*, were also correlated negatively with δ^13^C and positively with bark thickness. Bark thickness, in turn, was positively related with DBH and %LCC. (Fig 2c; S2b Table).

## Discussion

Our results showed that the three trait clusters (whole-plant, stem and leaf traits) are highly coordinated among them (Fig 2), reflecting a plant economics spectrum [29]. Although we found fewer and weaker covariations than we had expected, it is clear that traits are connected in a broad network. These results evidence that the existence of trade-offs organizing the diversity of possible plant phenotypes into individual trait clusters found across species and at large spatial scales, it is not maintained at the intraspecific level, at least for the study species.

Studies at the community level showed that trade-offs proposed by LES between acquisition and resources conservation are not always found [12,60–62], reflecting different sensitivity of LES traits to different scales depending on the main environmental, genetic and biophysical drivers affecting them [12]. Natural selection shapes phenotypes with correlated traits based on the interaction of genes and the environmental conditions. It means that certain trait combinations may be favoured or discarded in given environments [63,64]. At the intraspecific scale, evidence analysing this pattern is scarce and highly species-specific [6,65–68]. For example, it is remarkable the lack of relationships in our data between SLA and other leaf traits, like N content, to which has been frequently associated [69]. This lack of correlations can be explained by the coniferous nature of our study species, following the outcomes described by Lusk et al. [70], who showed no relationship between leaf N and SLA in other conifers, in a comprehensive study including many angiosperms and conifers. This result supports the idea that LDMC is a more reliable leaf trait to reflect the leaf trait cluster as suggested by Wilson et al. [71], especially for conifers, than SLA, which depends greatly on the spatial dimensions of leaves that are highly variable [72]. Similarly, we found remarkable the lack of correlation among photosynthetic pigments and other leaf traits, like N content, a pattern described in literature [73–75]. Looking at the variability of leaf traits (Table 2), we suggest that it might be associated with their low variability (all below 15%, excluding the pigments) compared to the other studied traits and findings from other studies addressed at the intraspecific level [30] that encompassed a wider environmental gradient.

The plant size traits were tightly correlated, being taller those trees with thicker trunks and larger crowns. This plant size cluster is complemented with stem traits, as bigger trees had denser wood and thicker bark. Wood density is considered a good indicator of mechanical resistance and hydraulic safety [76–79]. Namely, it is associated to the resistance to embolism, because the greater is the wood density, the narrower are the conduits that confer embolism resistance [80–82]. Cold and drought imperil plants to embolism, and plants in dry or cold areas usually have narrower water-conducting conduits compared to plants thriving in moist, warm areas. Thus, narrow conduits have been considered a key adaptation to cold and drought conditions, together with short plant height [82]. In the line with our results, other studies have found that bark thickness also correlates positively with tree size, bestowing trunk mechanical strength and protection against frost and drought [44,83–86]. Similarly, previous studies have shown the relationship between bark thickness and DBH [85,86] with increasing bark accumulation as trees grow.

Moreover, our results showed interactions among leaf traits and other trait clusters within a species, similarly to studies analysing simultaneously trait clusters in different species [19,21–23]. For instance, increasing LDMC and leaf C content appeared in bigger trees. Greater SLA was also connected with smaller trees with lower WD. Leaf traits were measured in needles collected from the top part of the crown that did not necessary meant fully exposed needles in dominated trees. Therefore, this correlation would reflect the effect of light availability (greater in taller trees) on leaf characteristics [87]. Altogether, this outcome supports our first prediction that all traits measured on leaves, stem and plant size co-vary shaping a broad network of coordinated trait clusters. In other words, they endorsed the existence of a whole-plant economics spectrum.

Our study showed a different pattern in the trait coordination in the two close sites in contrasting elevation, reflecting different sensitivity of traits and their covariation to environmental conditions. An elevation difference of 450 m entailed, amongst others, different rainfall and temperature values (annual mean temperature of 6.5°C vs 7.9 °C and mean annual precipitation of 1242 mm *vs*. 897 mm), which triggered significant differences in functional traits among pines from both sites. Individuals growing at higher elevation presented greater bark thickness than those at lower elevation (S1 Fig), potential response to harsher conditions in terms of snow, wind at the top of the mountain [84]. Additionally, individuals growing at the lower elevation had leaves with lower SLA and greater LDMC (S1 Fig). These leaf characteristics are frequently found in dry sites [88], indicating a conservative strategy to extend the life-span of expensive organs (needles) in poor or adverse environments. Nevertheless, low elevation trees had also a higher amount of photosynthetic pigments, indicative of higher productivity. The explanation of this apparent inconsistency may lie in the most challenging conditions that plants experience at high elevations, as a combination of high irradiance and low temperatures [39]. Coldness at high elevations inhibits enzymatic reactions reducing carbon absorption, without affecting light capture, triggering a protection mechanism, photoprotection, which eventually implies a reduction of the amount of chlorophylls [89,90].

Two possible factors can underlie these changes in trait variation between both sites (S1 Fig): phenotypic plasticity and local genetic variation. These two factors are not mutually exclusive and they probably act together to help plants to more effectively deal with the different environmental conditions [91]; however, with our data, we cannot discern them. At the site level, we observed that elevation, as a proxy of other environmental variables such temperature, vegetation and stoniness, accounted for a small but significant 5% of total trait variation. Indeed, trait variation (in CV) was greater at low elevation (Table 2) which suggests that trait variability decreases under more stressful conditions. An explanation would be a greater amount of available resources in mild sites that would provide more opportunities (i.e. increase the available niche space) to individuals, that could thrive in different micro-sites [5,7,38]. On the other hand, at high elevation we found a greater number of correlations between clusters revealing that pines up there displayed a more coordinated phenotype compared with those at low elevation. This entails that elevation, as a proxy of others environmental factors, played an important role in the trait coordination, detectable in close individuals of Scots pine. Moreover, despite gene flow is expected between both stands due to the anemophilous pollination of pines and the proximity between them (ca 7km), we acknowledge that genetic differences may partly explain the remaining unexplained variation for phenotypic differences between both sites. An integrated phenotype means that any change in one trait implies changes in the whole phenotypic response of an individual [36]. This scaling relationship can be the result of natural selection acting over individuals at the treeline [92], favouring the coordination and adjustment of the whole phenotype to the environmental conditions at such elevation. Our results agree with those hypothesised by Keddy [93] and Violle et al. [4] which posed that environmental factors act as filters at the individual level. On the contrary, individuals at low elevation did not show the same trait coordination, evidencing that changes in a given trait would not necessarily unleash shifts in traits from a different cluster.

This work represents one of the first attempts to analyse phenotypic coordination within a species under contrasting environmental conditions, in a comprehensive way with a large array of traits. The significant correlations among three trait clusters related to important dimensions of the global plant spectrum, revealed the existence of a more complex and coordinated phenotypes than those found in single-spectrum or one trait cluster approaches. In addition, our results showed differences in the trait correlation networks induced by contrasting environmental conditions that provide evidence for different sensitivity of traits and their covariation to the external conditions.

## Supporting information

S1 Appendix Detailed methods. (DOC)

S2 Appendix Descriptive statistics for the plant size traits measured on *Pinus sylvestris* individual trees. Confidence limits surrounding coefficients of variation (CV) were calculated through bootstrapping with replacement (with n = 500 replicates). Comparison of traits values: a) plant height; b) diameter at breast height (DBH) and c) Age at the two different elevations using a t-test are also shown.
(DOCX)

S1 Table Study traits, the ecological function they represent and literature describing them. (DOCX)

S2 Table Trait correlations between traits measured in a) pooled data of *Pinus sylvestris* and b) distinguishing the two study sites (under the diagonal trees from Pingarrón at 1900 m, and above the diagonal trees from Ventorrillo at 1440 m). It was used the Pearson´s rank correlation analyses. ‘ns’ means ‘not significant’. Brackets indicate the number of individuals at each correlation; number at left side is for Pingarrón correlations and at right side for Ventorrillo correlations.
(DOCX)

S1 Fig Bean plot of the traits that were significantly different between both elevations using a t-test (Pingarrón 1900m, Ventorrillo 1440 m). (SLA: specific leaf area; LDMC: leaf dry matter content).
(DOC)

S2 Fig Ordination diagram of traits (RDA) in relation to the elevation of both surveyed sites. The orientation of line directions indicates the sign of the correlation among the traits, and the length is related to the strength. Triangles indicate the centroid of both populations Pingarrón in pink (1900m) and Ventorrillo in green (1440m). (DBH: diameter at breast height; Stem WD: stem wood density; LDMC: leaf dry matter content; SLA: specific leaf area; LNC%: leaf nitrogen content, LCC%: leaf carbon content; δ^13^C leaf carbon isotope discrimination).
(DOC)
