# Supplementary material for: Intraspecific perspective of phenotypic coordination of functional traits in Scots pine
Source: PLoS One. 2020 Feb 13;15(2):e0228539. doi: 10.1371/journal.pone.0228539 (PMC7018023; doi:10.1371/journal.pone.0228539)
Supplement: S1 Appendix — (DOC) [file pone.0228539.s001.doc]

# s1 Appendix. Detailed methods

**Leaf Traits**

We followed the protocol suggested by Pérez-Harguindeguy *et al.* [1] for leaf trait assessment. In each tree, we collected a branch from the top part of the crown, i.e. exposed to the maximum light available for each individual and immersed it for 24 hours in water to rehydrate leaves. Then, we chose 10 healthy needles developed the previous season were chosen, weighed individually and scanned. Then they were dried in an oven at 60ºC for 72 hours and weighed again to obtain the dry weight. Leaf dry matter content (LDMC) was assessed as the ratio between the weight of a dried leaf (mg) and its saturated weight (g) (after rehydration). Specific Leaf Area (SLA) was calculated as the ratio between leaf area (mm^2^) and leaf dry mass (mg) of individual healthy leaves. Leaf Area was obtained analysing the images (scanning) by the Image Tool program 1.8.0. Leaf nitrogen content (LNC, %), leaf carbon content (LCC, %) and carbon isotope (δ^13^C), were calculated in the isotope lab of University of California, after grinding and encapsulating dried material. We followed the protocol described by García-Plazaola and Becerril [2] to extraction of the photosynthetic compounds (chlorophyll *a* and *b*, beta-carotene) using High Performance Liquid Chromatography (HPLC).

**Stem Traits**

Stem wood density (WD) was the ratio between wood dry mass (g) per displaced volume (g). We recorded the mass of rehydrated cores (for 24 hours) using a balance to the nearest 0.01 mm. Then, they were dried for 2 hours at 102 °C prior to measuring the volume based on water displacement method. The weight of the displaced water is equal to the volume of the sample with the equivalence 1 g = 1 cm^3^. Bark thickness was the mean of three bark measurements taken with a bark gauge per individual.

**Plant size**

Plant total height and crown depth were obtained in field with a clinometer. DBH was measured with a diametric tape.

**References**

1. **Pérez-Harguindeguy N, Diaz S, Garnier E, *et al.* 2013**. New Handbook for standardized measurment of plant functional traits worldwide. *Australian Journal of Botany* **61**:167–234.
2. **García-Plazaola JI, Becerril JM. 2001.** Seasonal changes in photosynthetic pigments and antioxidants in beech (*Fagus sylvatica*) in a Mediterranean climate: implications for tree decline diagnosis. *Australian Journal of Plant Physiology* **28**: 225–232.
