# Supplementary material for: Intraspecific perspective of phenotypic coordination of functional traits in Scots pine
Source: PLoS One. 2020 Feb 13;15(2):e0228539. doi: 10.1371/journal.pone.0228539 (PMC7018023; doi:10.1371/journal.pone.0228539)
Supplement: S2 Appendix — Confidence limits surrounding coefficients of variation (CV) were calculated through bootstrapping with replacement (with n = 500 replicates). Comparison of traits values: a) plant height; b) diameter at breast height (DBH) and c) Age at the two different elevations using a t-test are also shown. (DOCX) [file pone.0228539.s002.docx]

**S2 Appendix. Descriptive statistics for the plant size traits measured on *Pinus sylvestris* individual trees.** Confidence limits surrounding coefficients of variation (CV) were calculated through bootstrapping with replacement (with n = 500 replicates). Comparison of traits values: a) plant height; b) diameter at breast height (DBH) and c) Age at the two different elevations using a t-test are also shown.

|  | **Plant height** |  | **DBH** |  | **Age** |
| --- | --- | --- | --- | --- | --- |
| **Pingarrón (1900m)** |  |  |  |  |  |
| Range | 2.11 - 20.76 |  | 4.0 - 107.9 |  | 11 - 163 |
| Mean ± SD | 13.68 ± 3.93 |  | 33.89 ± 16.22 |  | 79 ± 35.2 |
| CV | 0.28 (0.26, 0.30) |  | 0.47 (0.44, 0.50) |  | 0.44 (0.41 - 0.43) |
|  |  |  |  |  |  |
| **Ventorrillo (1440m)** |  |  |  |  |  |
| Range | 3.61 - 20.08 |  | 0.53 - 71.0 |  | 33 - 145 |
| Mean ± SD | 12.49 ± 3.72 |  | 33.78 ± 18.11 |  | 72 ± 29.1 |
| CV | 0.29 (0.26, 0.30) |  | 0.53 (0.49, 0.56) |  | 0.40 (0.37 - 0.41) |
|  |  |  |  |  |  |
| *t*-value | 1.88 |  | 0.72 |  | 0.3 |
|  |  |  |  |  |  |
| **Pooled data** |  |  |  |  |  |
| Range | 2.11 - 20.76 |  | 0.53 - 107.9 |  | 11 - 163 |
| Mean ± SD | 12.91 ± 3.89 |  | 38.05 ± 20.34 |  | 75 ± 32 |
| CV | 0.29 (0.26, 0.31) |  | 0.50 (0.47, 0.53) |  | 0.42 ( 0.39 - 0.44) |

**
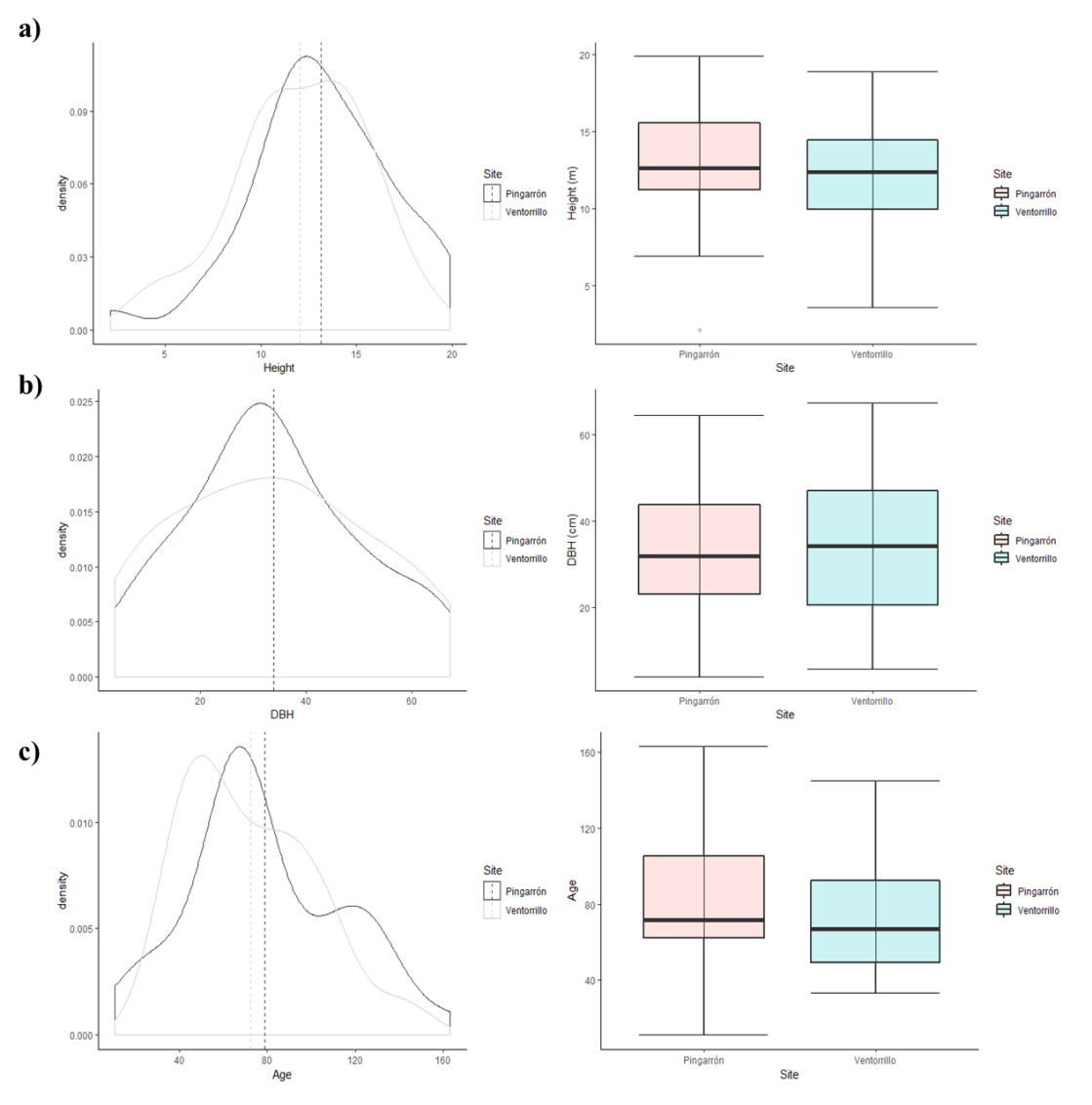
**
