# Supplementary material for: Intraspecific perspective of phenotypic coordination of functional traits in Scots pine
Source: PLoS One. 2020 Feb 13;15(2):e0228539. doi: 10.1371/journal.pone.0228539 (PMC7018023; doi:10.1371/journal.pone.0228539)
Supplement: S1 Table — (DOCX) [file pone.0228539.s003.docx]

# **S1 Table.** Study traits, the ecological function they represent and literature describing them.

| **Trait cluster** | **Trait** | **Ecological significance** | **Refs** |
| --- | --- | --- | --- |
| Plant size | Plant height | Competitive ability, photosynthetic behaviour, hydraulic limitations and probability of fire escape. | Gaudet and Keddy (1988); Rijkers et al. (2000); Niklas (2007); Higgins et al. (2012); |
|  | Crown depth | Magnitude of light capture, competitive vigour, tree growth performance. | Valladares and Ninemets (2007); Osada (2011); Osada and Hiura (2017); Li et al. (2017); Carvalho and Ribeiro (2018) |
|  | Diameter at breast height | Competitive vigour, whole plant fecundity, growth time between disturbances, photosynthetic behaviour and probability of fire escape. | Kenkel et al. (1997); Martínez-Vilalta et al. (2007) |
|  |  |  |  |
| Stem traits | Bark thickness | Trunk insulation against fire, pathogen, frost and drought, and trunk mechanical strength. | Vines (1968); Niklas (1999); Paine et al. (2010); Brando et al. (2011); Molina et al. (2016) |
|  | Stem wood density | Growth-survival trade-off, mechanical resistance, water storage in the trunk, net CO2 assimilation, hydraulic safety and response to precipitation and altitude. | Enquist et al. (1999); Santiago et al. (2004); Chave et al. (2006); van Gelder et al. (2006); Poorter et al. (2008); Sperry et al. (2008); Leoneli et al. (2009); Niklas and Spatz (2010) |
|  |  |  |  |
| Leaf traits  (Leaf morpho-anatomy) | Leaf dry matter content | Structural support of the leaf, anti-herbivory resistance, leaf tissue density, leaf life-span, relative growth rate of the plant. | Poorter and Bergkotte (1992); Niinemets and Kull (1995); Niinemets (1999); Niinemets (2001); Westoby et al. (2002); Kazakou et al. (2006); Poorter et al. (2009); Pérez‐Harguindeguy et al. (2013) |
|  | Specific leaf area | Resource acquisition, photosynthetic rate, relative growth rate of the plant, shade-tolerance. | Reich et al. (1992); Niinemets and Kull (1995); Cornelissen et al. (1996); Reich et al. (1997); Reich et al. (1999); Kazakou et al. (2006) |

| **Trait group** | **Trait** | **Ecological significance** | **Refs** |
| --- | --- | --- | --- |
| Leaf traits  (Leaf chemical composition) | Leaf nitrogen content | Net photosynthetic capacity, relative growth rate and N availability in the soil, leaf life-span and leaf decomposability. | Reich et al. (1992); Enriquez et al. (1993); Cornelissen et al. (1997); Reich et al. (1997); Reich et al. (1999); Niinemets et al. (2002); Martínez-Vilalta et al. (2007); Reich et al. (2008); Zhang et al. (2016) |
|  | Leaf carbon content | Leaf palatability, leaf lignin, leaf density, relative growth rates and structural leaf support. | Poorter and Bergkotte (1992); Niinemets et al. (2007) |
|  | Carbon isotope | Water use efficiency, the ratio of internal to atmospheric CO_2_ concentration, stomatal conductance, soil moisture, air temperature. | Warren et al. (2001); Van de Water et al. (2002); Martínez-Vilalta et al. (2007); Seibt et al. (2007); Zhang et al. (2016) |
|  | Chlorophyll a | Maximize net carbon gain; photosynthetic activity. | Krause and Weis (1991); Green and Durnford (1996) |
|  |  |  |  |
|  | Chlorophyll b | Maximize net carbon gain; photosynthetic activity. | Krause and Weis (1991); Green and Durnford (1996) |
|  |  |  |  |
|  | Beta-carotene | Maximize net carbon gain; photosynthetic activity. | Krause and Weis (1991); Green and Durnford (1996) |

**References**

**Arco Molina JG, Hadad MA, Patón Domínguez D, Roig FA. 2016.** Tree age and bark thickness as traits linked to frost ring probability on *Araucaria araucana* trees in northern Patagonia. *Dendrochronologia* **37**: 116–125.

**Brando PM, Nepstad DC, Balch JK, *et al.* 2012.** Fire-induced tree mortality in a neotropical forest: The roles of bark traits, tree size, wood density and fire behavior. *Global Change Biology* 18:630–641.

**Carvalho B, Ribeiro SP. 2018.** Architecture of *Mabea fistulifera* Mart. (Euphorbiaceae), a Neotropical semideciduous tree: development and variations in crown allometry between environments. *Flora* **239**:104–110.

**Chave J, Muller-Landau HC, Baker TR, *et al.* 2006.** Regional and phylogenetic variation of wood density across 2456 neotropical tree species. *Ecological Applications* **16**: 2356–2367.

**Cornelissen JHC, Werger MJA, Castro-Díez P, *et al.* 1997.** Foliar nutrients in relation to growth, allocation and leaf traits in seedlings of a wide range of woody plant species and types. *Oecologia* **111**: 460–469.

**Cornelissen JHC. 1996.** An experimental comparison of leaf decomposition rates in a wide range of temperate plant species and types. *Journal of Ecology* **84**: 573-582.

**Enquist BJ, West GB, Charnov EL, *et al.* 1999.** Allometric scaling of production and life history variation in vascular plants. *Nature* **401**: 907–911.

**Enriquez S, Duarte C, Sand-Jensen K. 1993.** Patterns in decomposition rates among photosynthetic organisms: the importance of detritus C: N: P content. *Oecologia* **94**: 457–471.

**Gaudet C, Keddy P. 1988.** A comparative approach to predicting competitive ability from plant traits. *Nature* **334**: 242–243.

**Green BR, Durnford DG. 1996**. The chlorophyll-carotenoid proteins of oxygenic photosynthesis. *Annual Review of Plant Physiology and Plant Molecular Biology* **47**: 685–714.

**Higgins SI, Bond WJ, Combrink H, *et al.* 2012.** Which traits determine shifts in the abundance of tree species in a fire-prone savanna? *Journal of Ecology* **100**:1400–1410.

**Kazakou E, Vile D, Shipley B, *et al.* 2006.** Co-variations in litter decomposition, leaf traits and plant growth in species from a Mediterranean old-field succession. *Functional Ecology* **20**: 21–30.

**Kenkel NC, Hendrie ML, Bella IE. 1997.** A long-term study of *Pinus banksiana* population dynamics. *Journal of Vegetation Science* **8**: 241–254.

**Krause H, Weis W. 1991.** Chlorophyll Fluorescence and Photosynthesis: The Basics. *Annual Review of Plant Physiology and Plant Molecular Biology* **42**: 313-349.

**Leonelli G, Pelfini M, Battipaglia G, Cherubini P. 2009.** Site-aspect influence on climate sensitivity over time of a high-altitude *Pinus cembra* tree-ring network. *Climatic Change* **96**: 185–201.

**Li Y, Kröber W, Bruelheide H, *et al.* 2017.** Crown and leaf traits as predictors of subtropical tree sapling growth rates. *Journal of Plant Ecology* **10**: 136–145.

**Martínez-Vilalta J, Cochard H, Mencuccini M, *et al.* 2009.** Hydraulic adjustment of Scots pine across Europe. *New Phytologist* **184:** 353–364.

**Martínez-Vilalta J, Vanderklein D, Mencuccini M. 2007**. Tree height and age-related decline in growth in Scots pine (*Pinus sylvestris* L.). *Oecologia*, **150**: 529–544

**Niinemets Ü, Kull K. 1994**. Leaf weight per area and leaf size of 85 Estonian woody species in relation to shade tolerance and light availability*. Forest Ecology and Management* **70**: 1–10.

**Niinemets Ü, Portsmuth A, Tena D, Tobias M, Matesanz S, Valladares F. 2007.** Do we underestimate the importance of leaf size in plant economics? Disproportional scaling of support costs within the spectrum of leaf physiognomy. *Annals of Botany* **100**: 283– 303.

**Niinemets Ü, Seufert G, Steinbrecher R, Tenhunen JD. 2002.** A model coupling foliar monoterpene emissions to leaf photosynthetic characteristics in Mediterranean evergreen *Quercus species*. *New Phytologist* **153**: 257–275.

**Niinemets Ü. 1999**. Components of leaf dry mass per area thickness and density- alter leaf photosynthetic capacity in reverse directions in woody plants. *New Phytologist* **144**: 35– 47.

**Niinemets Ü. 2001.** Global-scale climatic controls of leaf dry mass per area, density, and thickness in trees and shrubs. *Ecology* **82**:453–469.

**Niklas KJ, Spatz HC. 2010**. Worldwide correlations of mechanical properties and green wood density. *American Journal of Botany* **97**: 1587–1594.

**Niklas KJ. 1999.** The mechanical role of bark. *American Journal of Botany* **86**: 465–469.

**Niklas KJ. 2007**. Maximum plant height and the biophysical factors that limit it. *Tree Physiology* **27**:433-40.

**Osada N, Hiura T. 2017.** How is light interception efficiency related to shoot structure in tall canopy species? *Oecologia* **185**: 29–41.

**Osada N. 2011.** Height-dependent changes in shoot structure and tree allometry in relation to maximum height in four deciduous tree species. Functional Ecology **25**: 777–786.

**Paine CET, Stahl C, Courtois EA, Patiño S, Sarmiento C, Baraloto C. 2010.** Functional explanations for variation in bark thickness in tropical rain forest trees. *Functional Ecology* **24:** 1202–1210.

**Pérez-Harguindeguy N, Diaz S, Garnier E, *et al.* 2013**. New Handbook for standardized measurment of plant functional traits worldwide. *Australian Journal of Botany* **61:**167– 234.

**Poorter H, Bergkotte M. 1992.** Chemical composition of 24 wild species differing in relative growth rate. *Plant, Cell & Environment* **15**: 221–229.

**Poorter H, Niinemets Ü, Poorter L, *et al.* 2009.** Causes and consequences of variation in leaf mass per area (LMA): a meta-analysis. *New Phytologist* **182**: 565–588.

**Poorter L, Lianes E, Moreno-de las Heras M, Zavala MA. 2012.** Architecture of Iberian canopy tree species in relation to wood density, shade tolerance and climate. *Plant Ecology* **213**: 707-722.

**Poorter L, Wright S, Paz H, Ackerly D, Condit R, Ibarra-Manríquez G, *et al.* 2008**. Are functional traits good predictors of demographic rates? Evidence from five Neotropical forests. *Ecology* **89**: 1908–1920.

**Reich P, Walters M, Ellsworth D. 1992**. Leaf life-span in relation to leaf, plant, and stand characteristics among diverse ecosystems. *Ecological Monographs* **62**: 365–392

**Reich PB, Ellsworth DS, Walters MB, *et al.* 1999**. Generality of leaf trait relationships: a test across six biomes. *Ecology* **80**: 1955–1969.

**Reich PB, Tjoelker MG, Pregitzer KS, Wright IJ, Oleksyn J, Machado JL. 2008**. Scaling of respiration to nitrogen in leaves, stems and roots of higher land plants. *Ecology Letters* **11:** 793-801.

**Reich PB, Walters MB, Ellsworth DS. 1997**. From tropics to tundra: global convergence in plant functioning. Proceedings of the National Academy of Sciences **94**: 13730–13734.

**Rijkers T, Pons TL, Bongers F 2000.** The effect of tree height and light availability on photosynthetic leaf traits of four new tropical species differing in shade tolerance. Functional Ecology **14**: 77–86.

**Santiago LS, Goldstein G, Meinzer FC, *et al.* 2004.** Leaf photosynthetic traits scale with hydraulic conductivity and wood density in Panamanian forest canopy trees. *Oecologia* **140**: 543–550.

**Seibt U, Rajabi A, Griffiths H, Berry JA. 2008.** Carbon isotopes and water use efficiency: Sense and sensitivity. *Oecologia* **155**: 441–454.

**Sperry JS, Meinzer FC, McCulloh KA. 2008**. Safety and efficiency conflicts in hydraulic architecture: Scaling from tissues to trees. *Plant, Cell & Environment* **31**: 632–645.

**Valladares F, Niinemets Ü. 2007.** The architecture of plant crowns: from design rules to light capture and performance. In: Pugnaire F, Valladares F, eds. *Functional Plant Ecology*. Taylor and Francis, New York, 101–150.

**Van de Water PK, Leavitt SW, Betancourt JL. 2002.** Leaf δ^13^C variability with elevation, slope aspect, and precipitation in the southwest United States. *Oecologia* **132**: 332–343.

**van Gelder HA, Poorter L, Sterck FJ, *et al*. 2006.** Wood mechanics, allometry, and life- history variation in a tropical rain forest tree community. *New Phytologist* **171**: 367– 378.

**Vines RG. 1968**. Heat transfer through bark, and the resistance of trees to fire. *Australian Journal of Botany* **16**: 499–514.

**Warren CR, McGrath JF, Adams MA 2001.** Water availability and carbon isotope discrimination in conifers. *Oecologia* **127**: 476–486.

**Westoby M, Falster DS, Moles AT, *et al.* 2002.** Plant ecological strategies: some leading dimensions of variation between species. *Annual Review of Ecology, Evolution, and Systematics* **33**: 125–159.
