# Supplementary material for: Intraspecific perspective of phenotypic coordination of functional traits in Scots pine
Source: PLoS One. 2020 Feb 13;15(2):e0228539. doi: 10.1371/journal.pone.0228539 (PMC7018023; doi:10.1371/journal.pone.0228539)
Supplement: S2 Table — It was used the Pearson´s rank correlation analyses. ‘ns’ means ‘not significant’. Brackets indicate the number of individuals at each correlation; number at left side is for Pingarrón correlations and at right side for Ventorrillo correlations. (DOCX) [file pone.0228539.s004.docx]

**S2 Table: Trait correlations between traits measured in a) pooled data of *Pinus sylvestris* and b) distinguishing the two study populations (under the diagonal trees from Pingarrón at 1900 m, and above the diagonal trees from Ventorrillo at 1440 m).** It was used the Pearson´s rank correlation analyses. ‘ns’ means ‘not significant’. Brackets indicate the number of individuals at each correlation; number at left side is for Pingarrón correlations and at right side for Ventorrillo correlations.

| **a)** | **Plant height** | **DBH** | **Crown depth** | **Bark thickness** | **WD** | **LDMC** | **SLA** | **δ13C** | **%LCC** | **%LNC** | **Chlorophyll *a*** | **Chlorophyll *b*** | **Beta carotene** |
| --- | --- | --- | --- | --- | --- | --- | --- | --- | --- | --- | --- | --- | --- |
| **Plant height** | **1 (100)** |  |  |  |  |  |  |  |  |  |  |  |  |
| **DBH** | 0.65 (100) | **1 (100)** |  |  |  |  |  |  |  |  |  |  |  |
| **Crown depth** | 0.72 (100) | 0.5 (100) | **1 (100)** |  |  |  |  |  |  |  |  |  |  |
| **Bark thickness** | 0.65 (100) | 0.83 (100) | 0.38 (100) | **1 (100)** |  |  |  |  |  |  |  |  |  |
| **WD** | 0.34 (95) | 0.24 (95) | ns | 0.27 (95) | **1 (95)** |  |  |  |  |  |  |  |  |
| **LDMC** | 0.29 (95) | 0.23 (95) | ns | 0.22 (95) | ns | **1 (95)** |  |  |  |  |  |  |  |
| **SLA** | -0.21 (95) | ns | -0.23 (95) | ns | -0.29 (90) | -0.45 (95) | **1 (95)** |  |  |  |  |  |  |
| **δ13C** | ns | ns | ns | ns | ns | ns | -0.42 (95) | **1 (95)** |  |  |  |  |  |
| **%LCC** | ns | 0.27 (0.95) | ns | 0.34 (95) | ns | 0.25 (95) | ns | ns | **1 (95)** |  |  |  |  |
| **%LNC** | ns | ns | ns | ns | ns | ns | ns | ns | -0.25 (95) | **1 (95)** |  |  |  |
| **Chlorophyll *a*** | ns | ns | ns | ns | ns | ns | ns | -0.28 (95) | ns | ns | **1 (95)** |  |  |
| **Chlorophyll *b*** | ns | ns | ns | ns | ns | ns | ns | ns | ns | ns | 0.88 (95) | **1 (95)** |  |
| **Beta carotene** | ns | ns | ns | ns | ns | ns | ns | ns | ns | ns | 0.88 (95) | 0.87 (95) | **1 (95)** |
|  |  |  |  |  |  | **All** |  |  |  |  |  |  |  |

| **b)** | **Plant height** | **DBH** | **Crown depth** | **Bark thickness** | **WD** | **LDMC** | **SLA** | **δ13C** | **%LCC** | **%LNC** | **Chlorophyll *a*** | **Chlorophyll *b*** | **Beta carotene** |  |
| --- | --- | --- | --- | --- | --- | --- | --- | --- | --- | --- | --- | --- | --- | --- |
| **Plant height** | **1 (50)** | 0.72 (50) | 0.75 (50) | 0.64 (50) | ns | 0.29 (48) | ns | ns | ns | ns | ns | ns | ns | **Ventorrillo (1440 m)** |
| **DBH** | 0.58 (50) | **1 (50)** | 0.52 (50) | 0.83 (50) | 0.33 (47) | ns | ns | ns | 0.29 (48) | ns | ns | ns | ns |  |
| **Crown depth** | 0.75 (50) | 0.51 (50) | **1 (50)** | 0.31 (50) | ns | ns | ns | ns | ns | ns | ns | ns | ns |  |
| **Bark thickness** | 0.63 (50) | 0.84 (50) | 0.47 (50) | **1 (50)** | 0.36 (47) | ns | ns | 0.3 (48) | 0.39 (48) | ns | ns | ns | ns |  |
| **WD** | 0.43 (48) | ns | ns | ns | **1 (48/47)** | ns | ns | ns | ns | ns | ns | ns | ns |  |
| **LDMC** | 0.37 (47) | ns | 0.29 (47) | 0.31 (47) | ns | **1 (47/48)** | -0.33 (48) | ns | 0.52 (48) | ns | ns | ns | ns |  |
| **SLA** | -0.39 (47) | ns | -0.33 (47) | -0.34 (47) | ns | -0.55 (47) | **1 (47/48)** | -0.38 (48) | ns | ns | 0.36 (48) | ns | ns |  |
| **δ13C** | ns | ns | ns | ns | ns | ns | -0.49 (47) | **1 (47/48)** | ns | ns | -0.39 (48) | -0.29 (48) | ns |  |
| **%LCC** | ns | ns | ns | ns | ns | ns | ns | ns | **1 (47/48)** | -0.32 (48) | ns | ns | ns |  |
| **%LNC** | ns | ns | ns | ns | -0.35 (45) | ns | ns | ns | ns | **1 (47/48)** | ns | ns | ns |  |
| **Chlorophyll *a*** | ns | ns | ns | ns | ns | ns | ns | ns | ns | ns | **1 (47)** | 0.85 (48) | 0.87 (48) |  |
| **Chlorophyll *b*** | ns | ns | 0.33 (47) | ns | ns | ns | ns | ns | ns | ns | 0.93 (47) | **1 (47)** | 0.86 (48) |  |
| **Beta carotene** | 0.29 (47) | ns | ns | ns | ns | ns | ns | ns | ns | ns | 0.89 (47) | 0.86 (47) | **1 (47)** |  |
|  |  |  |  |  |  | **Pingarrón (1900 m)** | | |  |  |  |  |  |  |
