# Supplementary figures and images for: Intraspecific perspective of phenotypic coordination of functional traits in Scots pine
Source: PLoS One. 2020 Feb 13;15(2):e0228539. doi: 10.1371/journal.pone.0228539 (PMC7018023; doi:10.1371/journal.pone.0228539)

**S1 Fig.**

**
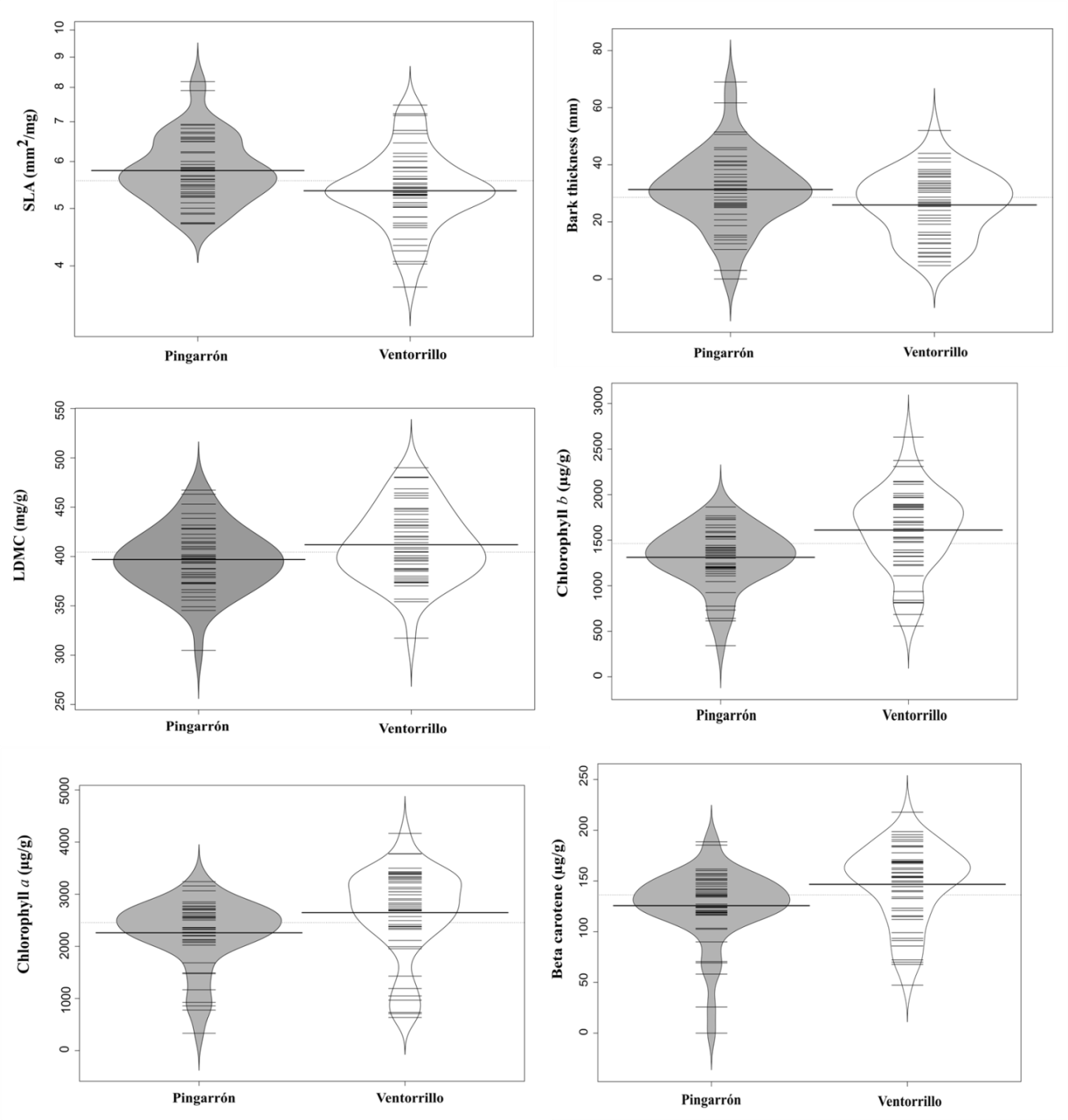
**

Supplement: S1 Fig — (SLA: specific leaf area; LDMC: leaf dry matter content). (DOC) [file pone.0228539.s005.doc]

**S2 Fig.**


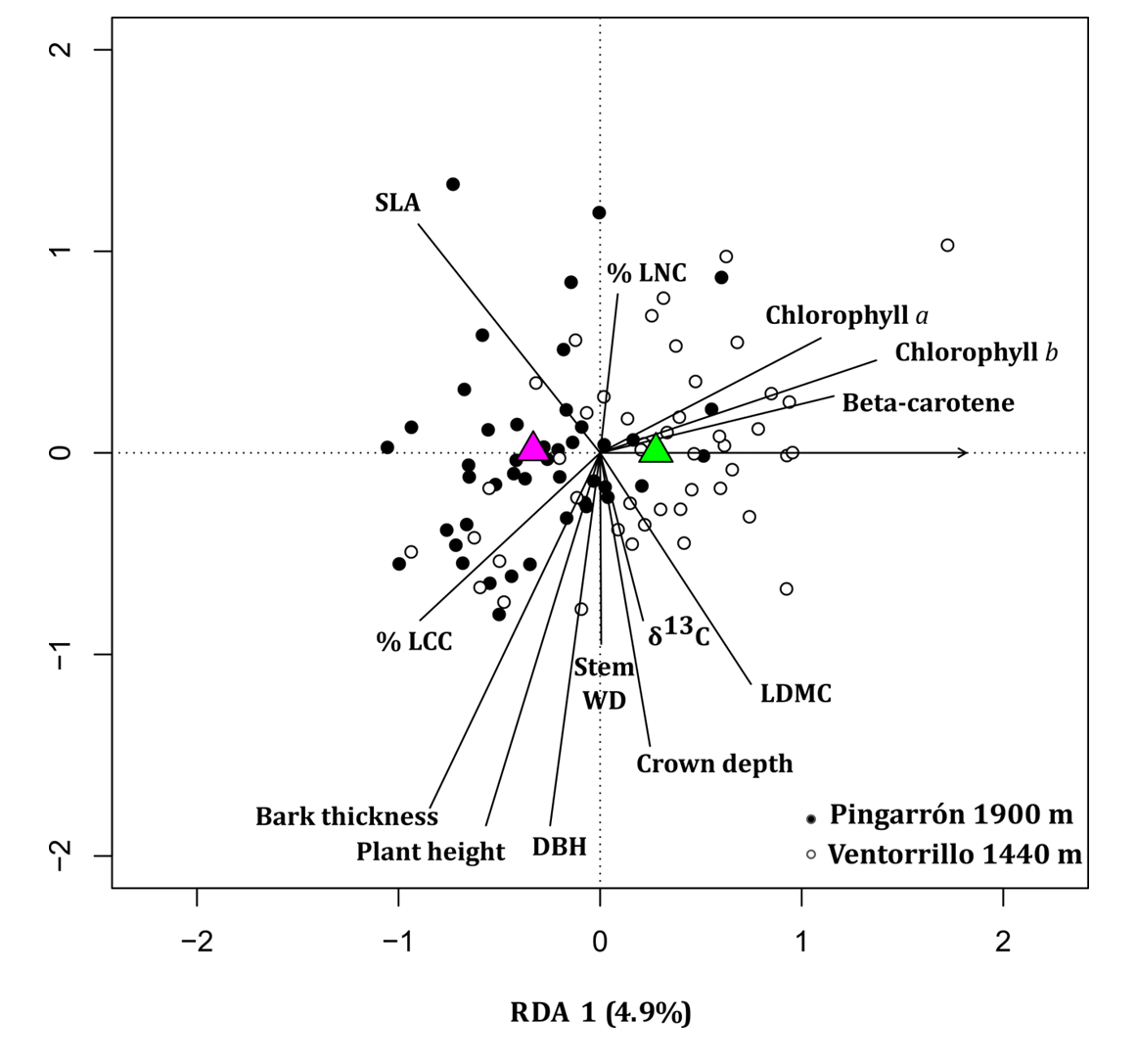

Supplement: S2 Fig — The orientation of line directions indicates the sign of the correlation among the traits, and the length is related to the strength. Triangles indicate the centroid of both populations Pingarrón in pink (1900m) and Ventorrillo in green (1440m). (DBH: diameter at breast height; Stem WD: stem wood density; LDMC: leaf dry matter content; SLA: specific leaf area; LNC%: leaf nitrogen content, LCC%: leaf carbon content; δ13C leaf carbon isotope discrimination). (DOC) [file pone.0228539.s006.doc]
